# Feasibility of medicinal cannabis cultivation in outdoor conditions using municipal reclaimed water

**DOI:** 10.1186/s42238-026-00433-9

**Published:** 2026-05-06

**Authors:** Tomás Bosco, Mariana Lozada, Mauricio Faleschini, Gregorio Bigatti

**Affiliations:** 1 Programa Interdisciplinario de Cannabis del CCT CONICET-CENPAT, Puerto Madryn, Chubut, Argentina; 2 Instituto Patagónico de Ecosistemas Continentales (IPEEC, CONICET), CCT CONICET- CENPAT, Puerto Madryn, Chubut Argentina; 3 Instituto de Biología de Organismos Marinos (IBIOMAR, CONICET), CCT CONICET-CENPAT, Puerto Madryn, Chubut Argentina; 4 Centro para el Estudio de Sistemas Marinos (CESIMAR, CONICET), CCT CONICET-CENPAT, Puerto Madryn, Chubut Argentina; 5 Universidad Espíritu Santo, Guayaquil, Ecuador

**Keywords:** Treated wastewater, Sustainable agriculture, Water quality, Yield, Cannabis safety

## Abstract

Increasing pressure on freshwater resources represents a major challenge for sustainable agricultural production in arid and semi-arid regions. In this context, the use of treated wastewater in agriculture may enhance environmental sustainability and economic efficiency. This study evaluated the yield and safety of outdoor cannabis cultivation irrigated with reclaimed domestic wastewater in an arid Patagonian environment (Argentina). Key metrics included soil and water quality, flower and cannabinoid yield, and chemical and microbiological safety. Soil and water quality were adequate in terms of physicochemical parameters. *Escherichia coli* in irrigation water at the time of tank filling was 1,700 ± 733 MPN/100 mL. Irrigation was initiated at these concentrations, and *E. coli* levels declined during subsequent storage in tanks, reaching values below 1,000 MPN/100 mL after approximately 10 days, consistent with the World Health Organization guideline for unrestricted agricultural reuse. *E. coli* and total coliforms were absent in leaves, flowers, and derived products (herbal oils). Potentially hazardous heavy metals (As, Cd, Co, Cr, Cu, Ni, Pb, and Zn) did not show systematic accumulation in the system. Concentrations in flowers and oils were generally below regulatory standards for edibles; however, Pb levels in dried flowers exceeded established regulatory limits. Dried flower yields were approximately 267.77 ± 34.99 g/plant. Cannabinoid concentrations ranged from 7–14%, comparable to values previously reported for these varieties when cultivated indoors. Overall, the results support the potential of reclaimed wastewater as an irrigation alternative in arid regions, although continued monitoring of contaminants is essential to ensure product safety and regulatory compliance.

## Introduction

The agricultural reuse of treated wastewater addresses critical global challenges related to water scarcity, particularly in arid and semi-arid regions. Treated wastewater generally refers to municipal or domestic effluents that have undergone primary and secondary treatment processes, and in some cases tertiary treatment, to reduce organic matter, nutrients, and microbial loads prior to reuse (National Research Council 1996). Among the different wastewater sources available, municipal treated wastewater is most commonly considered for agricultural irrigation due to its relatively stable composition and established regulatory frameworks (Yalin et al. 2023). As urban populations expand, the demand for water increases, intensifying pressure on already limited freshwater resources (Tran et al. 2016; Jones et al. 2024). Currently, more than 40% of the global population lives in regions affected by water scarcity, and global water demand is projected to increase by 20–30% by 2050, largely driven by agricultural and urban uses (FAO 2020; UNESCO 2023). Agriculture, consuming approximately 70% of the world’s freshwater, represents the most significant strain on these resources (Aivazidou et al. 2016; Mialyk et al. 2024). Employing treated wastewater as an alternative irrigation source reduces this pressure, contributing to environmental conservation and the sustainability of agricultural practices (Pescod 1992; Zhao et al. 2022). Guidelines developed by the World Health Organization (WHO) and the Food and Agriculture Organization (FAO) outline the conditions under which treated wastewater can be safely utilized. These guidelines emphasize the importance of monitoring for potential risks, including salinity issues, heavy metals levels, and microbial contamination, which could affect soil health, crop safety, and ultimately human health (WHO 2006, 2018; Dreschel et al. 2022; Zhao et al. 2022; Qadir et al. 2025).

*Cannabis sativa* L. has gained prominence in recent years for its industrial and medicinal applications, as more countries started to revise their previously restrictive legislation in response to its health and economic potential. Certain cannabis cultivars are particularly valued for their high fiber and seed yield (Crini et al. 2020), while others classified as “medicinal cannabis” have high production of bioactive compounds, including the cannabinoids tetrahydrocannabinol (THC) and cannabidiol (CBD). Cannabinoids possess therapeutic properties for diverse conditions such as chronic pain, epilepsy, certain tumors and anxiety (Kisková et al. 2019; Sarris et al. 2020; Longo et al. 2021; Hameed et al. 2023; Borowicz et al. 2024; Devinsky et al. 2024). The cultivation of cannabis therefore presents an opportunity to integrate sustainable irrigation practices, such as the use of treated wastewater, into high-value crop production. Despite its benefits, this approach needs a rigorous evaluation of plant health, soil quality, and product safety to ensure compliance with agricultural and public health standards (WHO 2007). The importance of crop irrigation with wastewater has been recognized as a promising strategy to mitigate desertification (Barbosa et al. 2015; Al Hamedi et al. 2023). Despite its potential use, little work has been done focusing on the evaluation of cannabis cultivars irrigated with reclaimed water (Mendel et al. 2022).

Puerto Madryn, a small city with 102,000 inhabitants located in Chubut province, in the arid Patagonian region of Argentina, has a municipal sewage treatment system consisting of 50 ha of natural stabilization ponds known as “Cota 130”, with a design depth of 1.5 m and a treatment capacity of around 20,000 m^3^ day⁻^1^. This system has a long history of reclaimed water reuse for the irrigation of agricultural crops (Faleschini 2016). The use of treated municipal wastewater for these activities reflects the regional hydrological context and productive constraints of northeastern Patagonia, where potable water is supplied from the Chubut River, located approximately 60 km from the city. Under these conditions, the use of potable water for agricultural irrigation at a productive scale is technically, ecologically, and economically unfeasible. In this context, access to treated municipal wastewater enables agricultural activities such as the production of olive trees, capers, fine fruits, alfalfa, and forest tree species that would otherwise not be possible under local water availability conditions. Additionally, this practice avoids the discharge of treated water into the coastal area adjacent to Península Valdés, a UNESCO World Heritage Site (UNESCO 1999), which hosts high biological diversity, including marine mammal species and hundreds of bird species that depend on wetlands of International Importance under the Ramsar Convention (Baldi et al. 2017; D’Agostino et al. 2023; Jiménez-Ramos et al. 2025).

In the last years, considerable efforts have been made in Argentina to develop a framework for both fiber and medicinal cannabis cultivation, culminating in the enactment of a provincial law regulating this activity in Chubut (Law N°790/24) in November 2024. Within this regulatory framework and as part of the development of a local cannabis research and industry sector, a breeding program was established in the Patagonian National Research Center (CCT CONICET-CENPAT), belonging to the Argentinean National Research Council (CONICET), resulting in the stabilization of six *Cannabis sativa* L. cultivars with contrasting chemotypes, suitable for medicinal purposes and adapted to the extreme environmental conditions of northeastern Patagonia. These cultivars have been registered at the National Seed Institute (INASE), and evaluated under both indoor and outdoor commercial production systems in the region, with plantations exceeding 2,000 plants. In this framework, non-conventional water sources for agriculture were considered to mitigate environmental impacts and guarantee production in a zone where the commercial cultivation with fresh water is unviable. In contrast to previous studies focused on conventional crops, this study evaluates the feasibility and safety of cultivating *Cannabis sativa* L. for medical purposes using municipal reclaimed water under outdoor conditions in an arid environment, integrating agronomic performance, soil and water quality, and product safety.

## Methodology

### Reclaimed water source and treatment system

The water used for irrigation corresponded to treated municipal wastewater from the city of Puerto Madryn (Chubut Province, Argentina (42°47′10.4″ S, 65°00′28.2″ W; 5 m a.s.l.). Domestic wastewater (approximately 20,000 m^3^/day) is collected by the sewer network (covering about 80% of the population) and initially directed to a primary treatment using screens and sieves, before being pumped to the secondary treatment. This system is operated by the Municipality, based on stabilization ponds, without mechanical aeration. It comprises two facultative lagoons connected in series, both 1.5 m deep, followed by a maturation lagoon 1 m deep designed to improve microbiological quality and for the reserve of treated water in the colder months, with lower irrigation demand.

To achieve effluents suitable for agricultural reuse, wastewater entering the stabilization ponds undergoes a minimum hydraulic retention time of approximately 50 days, a design parameter intended to reduce pathogen loads toward levels recommended for agricultural reuse (e.g., < 1,000 MPN/100 mL). The treated effluent is pumped from the outlet channel of the lagoon system to agricultural reuse projects, where storage reservoirs have been built, for subsequent distribution to irrigators. This treatment configuration has been implemented for agricultural reuse in Puerto Madryn, as documented in regional technical reports and field studies (Faleschini 2016; Cremona et al. 2023; Parra et al. 2025), including its application for the irrigation of alfalfa, natural grasslands, and *Olea europaea* (Arbequina) under Patagonian steppe conditions.

### Experimental site and plant material

To evaluate the effects of municipal treated wastewater on the agronomic performance and safety of medicinal cannabis production, a field experiment was conducted using registered *Cannabis sativa* L. cultivars at the facilities of the Interdisciplinary Cannabis Program (PICANN) of CCT CONICET–CENPAT (42°47′12.1"S 65°00′30.2"W), located in Puerto Madryn city.

Puerto Madryn has a cold arid steppe climate (BSk, Köppen–Geiger classification), with a mean annual temperature of 14 °C, high thermal amplitude (up to 40 °C), and low annual precipitation, typically below 250 mm (Kottek et al. 2006; Climate-Data.org 2024). Vegetation is dominated by evergreen shrubs and perennial grasses adapted to the windy Patagonian environment (Muñoz et al. 2024).

During the experimental period (December 2021–March 2022), mean monthly temperatures ranged from 16.0 to 20.8 °C, with absolute maximum and minimum temperatures of 38.0 °C and 2.8 °C, respectively. Monthly precipitation varied between 2 and 30 mm, with up to six rainy days per month, remaining within the expected seasonal range for the region. Mean relative humidity ranged from 44 to 51%. Daylength decreased progressively from 13.4 h in December to 10.3 h in March, providing the natural photoperiodic conditions for flowering induction at the study site (Table 1).

**Table 1:** Monthly climatic conditions during the experimental period (December 2021–March 2022) at the study site in northeastern Patagonia. Values include mean, maximum and minimum air temperature (°C), total monthly precipitation (mm), mean relative humidity (%), and daylength (h)

Study period			Mean temperature (°C)	Maximum temperature (°C)	Minimum temperature (°C)	Total precipitation (mm)	Relative humidity (%)	Daylength (h)
Year	2021	Dicember	20.8	37.4	6.2	18	48	13.4
	2022	January	21	38	9.4	26	44	13.1
		Febraury	19	35.7	8	30	51	11.6
		March	16	31.4	2.8	2	46	10.3

Data source: Climatic data were obtained from the Trelew Airport meteorological station (RP5 Weather Archive 2022). Daylength values were calculated based on site latitude

The *Cannabis sativa* L. cultivars used in this study were developed by the PICANN and registered by CONICET at the National Seed Institute (INASE, Argentina). The selected cultivars are, ‘Conicet’, ‘Mariquita’, and ‘Pachamama’, and represent three distinct chemotypes: Type I (THC-dominant), Type II (CBD-THC balanced), and Type III (CBD-dominant) (Table 2).

**Table 2:** Main characteristics of *Cannabis sativa* L. cultivars used in this work. Cannabinoid concentrations (THC: Δ⁹-tetrahydrocannabinol; CBD: cannabidiol) correspond to official values reported during cultivar registration at the National Seed Institute (INASE, Argentina), based on standardized descriptors and determined under indoor cultivation conditions

Cultivar	CBD (%)	THC (%)	Major terpenes	Ratio _CBD:THC_	Chemotype
*Conicet*	0.3	10.4	α-pinene 30%; β- caryophyllene 25%; β-pinene12%; α-humulene 11%	-	I
*Mariquita*	6.7	2.4	(-)-guaiol 45%; β-caryophyllene 17%; (-)-α-bisabolol 13%	2.8:1	II
*Pachamama*	16.1	0.96	(-)-α-bisabolol 29%; (-)-guaiol 21%; β- caryophyllene 17%; myrcene 13%	16:1	III

All plants used in the experiment were obtained by clonal propagation from mother plants, following a standardized protocol (Caplan et al. 2018) under controlled indoor conditions at the CENPAT facilities. For each cultivar, 15 clones were produced. After 20 days, 10 clones per cultivar were selected to ensure uniformity at transplanting. Selection was based on visible rooting development (presence of well-formed roots and absence of deformities) and overall plant vigor, assessed visually to obtain homogeneous experimental units within each cultivar.

### Experimental design and crop management

The Selected clones were transplanted into 1 L pots containing commercial substrate and maintained under greenhouse conditions for a 10-day acclimatization period before being transferred outdoors (Wang et al. 2009; Caplan et al. 2023). Prior to outdoor transplantation, plant height (mean ± SE) was 26.10 ± 0.43 cm for ‘Pachamama’, 25.80 ± 0.27 cm for ‘Mariquita’, and 26.90 ± 0.17 cm for ‘Conicet’. The number of nodes on the main stem was 9.00 ± 0.26, 8.00 ± 0.26, and 9.00 ± 0.39, respectively. Before outdoor establishment, all plants were subjected to a uniform structural pruning, removing lateral branches and leaves from the basal 10 cm of the main stem. This practice was applied to improve plant architecture and to prevent contact between plant tissues, soil, and irrigation water.

After acclimatization, all plants were transplanted into an outdoor experimental plot surrounded by native vegetation and representative of the agroclimatic conditions of the study area. The soil consisted of locally sourced fill material with a sandy-loam texture. The plot was prepared with this material to ensure consistency with regional agricultural conditions. This practice is common in the study area, where productive fields are frequently established on recently filled soils using materials with improved fertility. Consequently, the experimental plot did not represent a stabilized pedogenetic soil profile. For contextual purposes, reference values for soils commonly used as filling material in the region, based on historical records from the Soil and Water Laboratory of IPEEC–CONICET, indicate organic carbon contents of 5.22 ± 0.36 g kg⁻^1^ (Walkley and Black), total nitrogen of 0.62 ± 0.06 g kg⁻^1^ (Kjeldahl), and available phosphorus of 5.84 ± 1.47 mg kg⁻^1^ (Olsen), with all soil analyses performed according to Burt & Soil Survey Staff (2014). These values are provided as indicative regional background information and do not represent site-specific measurements.

Plants were arranged in rows with spacing of 1 m between rows and 0.8 m between plants, resulting in an average planting density of 1.25 plants m^−2^. This configuration allowed for adequate plant growth, air circulation, and sunlight exposure. The experimental layout followed a systematic alternating arrangement of cultivars along each row to minimize positional effects. Each row consisted of three consecutive plants of each cultivar, followed by a fourth plant repeating the first cultivar in the sequence. Three rows were established, and each row began with a different cultivar, ensuring an even spatial distribution of the three cultivars across the experimental plot.

Plants were transplanted to the final plot on December 27, 2021, and harvest was completed on March 28, 2022. Due to local climatic conditions, the crop followed a short cycle of 78 to 90 days, comprising approximately 40 days of vegetative growth followed by a flowering period. Harvest occurred at different times due to maturity differences among cultivars: ‘Mariquita*’* and ‘Pachamama’ were harvested 78 days after transplanting, whereas ‘Conicet’ was harvested after 90 days. All inflorescences were harvested manually. To mitigate the effects of the predominant westerly winds in the region, which frequently generate gusts exceeding 50 km h⁻^1^, windbreaks made of transparent plastic sheets (20 cm height) were installed on the western side of each plant. Windbreaks and support structures were installed to reduce mechanical stress caused by these winds.

### Irrigation management

Plants were irrigated exclusively with municipal treated wastewater described in Sect. "Reclaimed water source and treatment system", starting immediately after transplanting into the outdoor experimental plot. Irrigation was applied using an automatic drip system equipped with self-compensating drippers, at a constant rate of 5 L per plant per day throughout the cultivation period. Considering the regional productive conditions, in which the use of potable water for agricultural irrigation at a productive scale is not viable, no potable-water control treatment was included. Therefore, the experimental design reflects realistic agricultural practices under local water availability constraints.

Reclaimed water was transported by tanker truck from the municipal wastewater treatment system and discharged into three irrigation tanks of 2,750 L each, connected to the irrigation system. A total of three tank refills were made during the cultivation cycle, approximately every 30 days. In all refilling events, the three irrigation tanks (2,750 L each) were filled simultaneously, and irrigation commenced one day after refilling. This waiting period was applied to allow sedimentation of suspended solids and sludge, reducing the risk of clogging in the drip irrigation system.

### Sample collection and analytical methods

For characterization of physicochemical and microbiological properties, soil samples were collected from the 0–10 cm layer at the beginning of the experiment, at 40 days, and at harvest. At each sampling time, five subsamples were taken from areas near the plants, within the irrigation influence zone, and then pooled into a single composite sample for analysis. For water characterization, samples were collected at each irrigation tank refilling event. For the first two refills, samples were taken at the time of refilling and 10 days afterward. In the third refilling event, an additional intermediate sampling was included 3 days after refilling, in addition to sampling at refilling and after 30 days. This modification was implemented in response to a higher initial *E. coli* concentration detected during the third refill, in order to better capture the early-stage microbial decay during storage.

After harvesting, inflorescences were dried under controlled conditions of humidity (55% relative humidity), light (darkness), temperature (15–18 °C) and constant aeration until constant weight. Once dried, cannabis oils were prepared following protocols developed by the Interdisciplinary Cannabis Program of CCT CENPAT–CONICET based on ethanol extraction methods for cannabinoids (Ramirez et al. 2019; Aragon et al. 2025). For each cultivar, a single composite sample was prepared by pooling inflorescences from the main stem of each of the ten plants, mixed in equal proportion to obtain a representative sample at the variety level. The procedure followed an ethanol-based extraction method described by Aragon et al. (2025), with minor modifications in batch size and final dilution volume. Briefly, 40 g of dried flowers were subjected to ethanol extraction, followed by concentration of the extract via rotary evaporation. The resulting resin was resuspended in extra virgin olive oil and diluted to a final volume of 360 mL. A single extraction was performed for each cultivar.

### Analyses

#### Physicochemical analyses

Soil texture was determined by the pipette method (Gee and Or 2002) at the beginning of the experiment, while pH and electrical conductivity (EC) were analyzed on the previously described sampling dates. Electrical conductivity was measured in a 1:5 soil–water extract using a portable electrical conductivity analyzer (ALTRONIX), and pH was measured potentiometrically in a 1:2.5 soil–water extract, using a pH meter (Orion 720 A) equipped with an automatic temperature compensation probe (U.S. Salinity Laboratory Staff method 1954). Water parameters including temperature, EC, pH, total dissolved solids (TDS), redox potential, and dissolved oxygen (DO), were measured at all sampling events using a multiparameter instrument (YSI-556). Water samples were also analyzed for total and volatile suspended solids, COD, BOD_5_, ammonium (NH_4_^+^), nitrite (NO_2_^−^), nitrate (NO_3_^−^) and phosphate (PO_4_^3−^) using standard methods (APHA 2017).

Soil, water samples (at the beginning of the experiment), harvested inflorescences, and the medicinal cannabis oil (after laboratory preparation) were analyzed for elemental concentrations of arsenic (As), cadmium (Cd), cobalt (Co), chromium (Cr), copper (Cu), nickel (Ni), lead (Pb), and zinc (Zn) using inductively coupled plasma atomic emission spectroscopy (ICP-AES).

#### Microbiological analyses

Microbial safety was assessed by analyzing the concentration of total coliforms and *Escherichia coli* in water, soil, dried inflorescences, and the final cannabis oil. Soil samples were analyzed only at the first (at transplanting) and the last (after 91 days) sampling dates. Microbial dynamics in the irrigation water were evaluated at each sampling event, as previously described. Inflorescences were analyzed prior to post-harvest drying, and the final oil extract was assessed after the production process. For inflorescence analyses, a composite sample was prepared for each cultivar by pooling the main stem inflorescences collected from the 10 plants per cultivar. From each composite sample, two subsamples (approximately 3 g each) were taken and analyzed independently for microbiological determination.

All samples were processed by homogenization and plated as appropriate. The "Most Probable Number" (MPN) technique was employed for microbial quantification, using Colilert test and Quanti-Tray of Idexx Laboratories, according to U.S. Environmental Protection Agency Method 1604 (2002).

#### Flower yield

Dry flower yield was calculated after the drying process as the total dry flower biomass per plant, expressed in grams.

#### Cannabinoid Content and cannabinoid yield

Flowers were analyzed for THC and CBD concentrations using two chromatographic methods: thin-layer chromatography (TLC) for initial screening, and gas chromatography with flame ionization detection (GC-FID) for precise quantification. Flower samples were homogenized and prepared using an ethanol-based extraction before analysis. For each cultivar, cannabinoid analyses were conducted on a homogenized composite sample obtained by pooling inflorescences from the main stems of the 10 plants. Cannabinoid content in flowers was calculated as mg/gr of dried flowers, where cannabinoid concentrations were estimated as the sum of THC + THCa + CBD + CBDa + CBN by High Pressure Liquid Cromatography (HPLC). Cannabinoids diluted in oils were quantified in the same way as above. Yield was calculated as mg/ml in oil and then referred to mg/g of dried flowers. Extraction efficiency (%) was calculated as yield oil/cannabinoid content in flowers * 100 (Rochfort et al. 2020).

## Results

### Soil physicochemical and microbiological properties

The soil was classified as sandy loam, with 58% sand, 33% silt, and 11% clay. Electrical conductivity (EC) values ranged between 200 and 312 μS/cm and pH remained stable throughout the crop cycle, with moderately alkaline values (7.4–7.5). Concentrations of heavy metals (As, Cd, Co, Cr, Cu, Ni, Pb, and Zn), remained stable throughout the cultivation period, with only minor fluctuations. Microbiological analyses of soil samples showed that Total coliform counts ranged from non-detectable levels at the beginning of the experiment to 9 MPN/g at harvest, while *E. coli* was not detected in either of the two sampling events (Table 3).

**Table 3:** Soil heavy metal concentrations (As: arsenic; Cd: cadmium; Co: cobalt; Cr: chromium; Cu: copper; Ni: nickel; Pb: lead; Zn: zinc) and microbiological characteristics (coliforms and *Escherichia coli*). Loq: limit of quantification (1 MPN/g for microbiological parameters; 0.20 mg/kg for heavy metals). MPN/g: most probable number per gram. ND: not determined

Time (days)	pH	EC (μS/cm)	Total coliforms (MPN/g)	*E.coli* (MPN/g)	Heavy metals (mg/kg)							
					As	Cd	Co	Cr	Cu	Ni	Pb	Zn
0	7.4	200	< Loq	< Loq	2.6	0.4	5.3	7.4	12.4	8.0	5.8	26.7
40	7.5	320	ND	ND	2.21	0.4	5.6	7.7	12.8	8.1	5.5	26.4
91	7.5	312	9	< Loq	2.4	0.4	5.4	8.1	12.8	8.0	5.5	26.7

### Water quality

The concentrations of metals in water samples were, in all cases, below the quantification limit of the method (< 0.002 mg/L), except for copper (Cu) and zinc (Zn), which were detected at very low levels (0.035 ± 0.001 and 0.116 ± 0.01 mg/L; respectively).

**Fig. 1:**
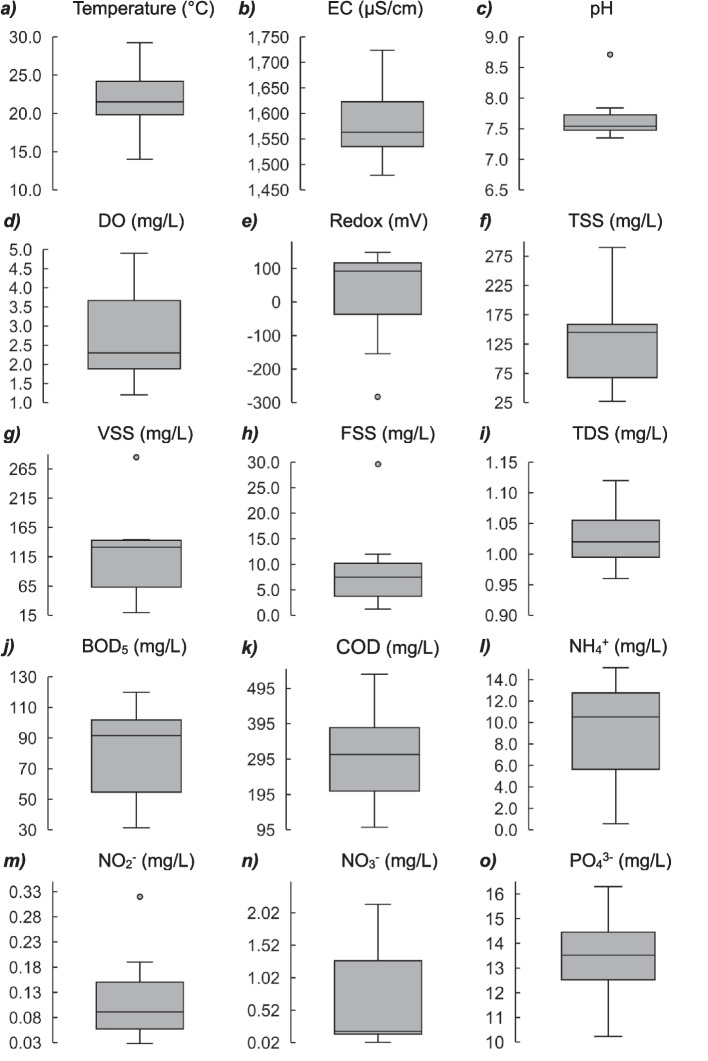
Mean values of physicochemical parameters: **a** temperature; **b** electrical conductivity (EC); **c** pH; **d** dissolved oxygen (DO); **e** redox potential (redox); **f** total suspended solids (TSS); **g** volatile suspended solids (VSS); **h** fixed suspended solids (FSS); **i** total dissolved solids (TDS); **j** biological oxygen demand (5 days; BOD_5_); **k** chemical oxygen demand (COD); **l** ammonium (NH_4_^+^); **m** nitrite (NO_2_^−^); **n** nitrate (NO_3_^−^); **o** and phosphate (PO_4_^3−^)) of reclaimed water used in this work

Reclaimed water showed mean pH values in the neutral range (7.71 ± 0.18), a mean temperature of 22 ± 5ºC and mean EC values of 1,583 ± 84 μS/cm (Fig. 1). Mean DO values were relatively low (2.8 ± 1.5 mg/L, range 1.2 to 4.9), while redox potential varied between + 148 and −283 mV. Biodegradable organic matter (BOD_5_) was present in the range of 30 to 120 mg/L, while COD values ranged from 102 to 535 mg/L. The total suspended solids were mainly represented by volatile material, associated with the presence of organic matter (measured as both BOD_5_ and COD). Regarding inorganic nutrients, nitrogen was mainly represented by NH_4_^+^ (10 ± 6 mg/L) and to a lesser extent by oxidized forms like NO_3_^−^ or NO_2_^−^. Phosphate (PO_4_^3−^) presented mean values of 13.43 ± 0.75 mg/L.

**Fig. 2:**
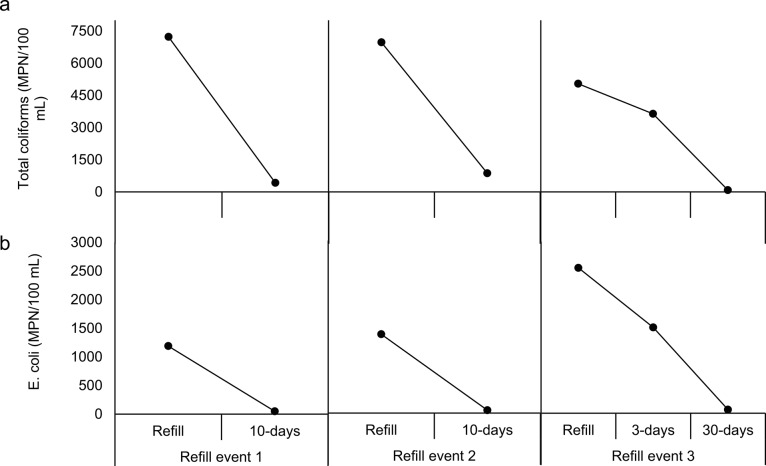
**a** Total coliforms and **b** *E. coli* abundances in reclaimed water as a function of storage time

Both total coliforms levels and *E. coli* counts decreased significantly with time of storage in the tanks. Total coliform counts in the first two refills were 7,225 and 6,970 MPN/100 mL at the time of refilling and decreased to 420 and 870 MPN/100 mL, respectively, after 10 days of storage. In the third refilling, initial counts were lower (5,040 MPN/100 mL), but the reduction was slower, with values of 3,640 MPN/100 mL after 3 days and 84 MPN/100 mL after 30 days (Fig. 2a). On the other hand, *E. coli* concentrations ranged from 1,190 to 1,370 MPN/100 mL in the first two refills, and decreased to values below 100 MPN/100 mL after 10 days of storage. In the third refilling, initial concentrations were slightly higher (2,540 MPN/100 mL), decreasing to 1,500 MPN/100 mL after 3 days, and reaching 62 MPN/100 mL after 30 days of storage (Fig. 2b).

**Fig. 3:**
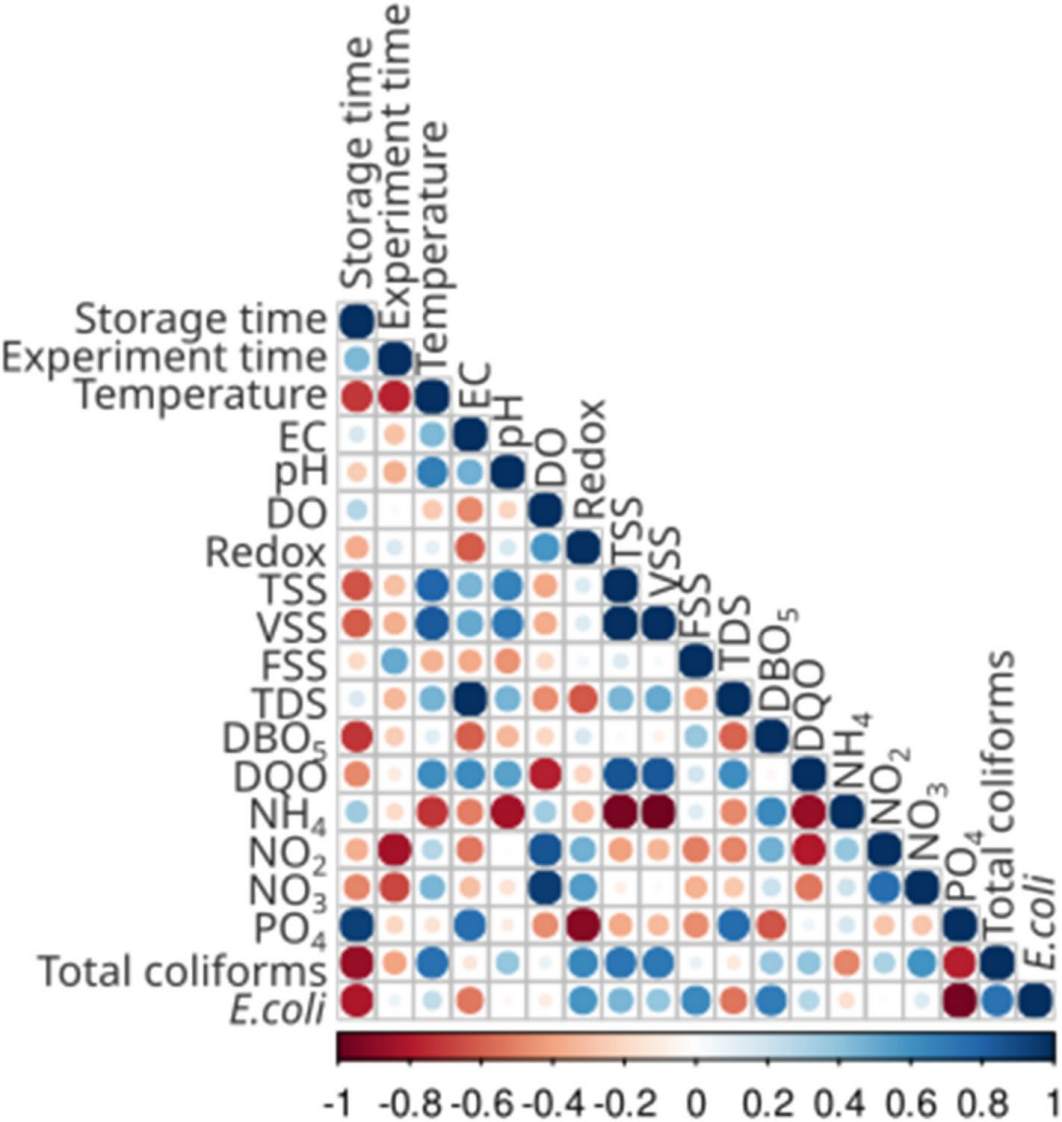
Correlation between treated water microbiological (Total coliforms and *E. coli*) and chemical parameters (temperature, electrical conductivity (EC), pH, dissolved oxygen (DO), redox potential (Redox), total suspended solids (TSS), volatile suspended solids (VSS), fixed suspended solids (FSS), total dissolved solids (TDS), biological oxygen demand (5 days; BOD_5_), chemical oxygen demand (COD), ammonium (NH_4_^+^), nitrite (NO_2_^−^), nitrate (NO_3_^−^), and phosphate (PO_4_^3−^)) in reclaimed water used in this work. Experiment Time: total time after the experiment start. Storage time: Time after the last refilling. Scale: Blue color indicates positive correlation and red color indicates negative correlation. The color intensity and size of the circles is proportional to the correlation coefficient (Pearson`s product-moment correlation)

Moderate positive correlation with some physicochemical variables was observed, namely total and volatile suspended solids, redox potential, BOD_5_ and negative with PO_4_^3−^. In addition, *E. coli* was positively correlated with FSS, and total coliforms were positively correlated with NO_3_^−^ and negatively correlated with NH_4_^+^ (Fig. 3).

### Flower yield and cannabinoid profiles

The average dry flower yield per plant was 267.77 ± 34.99 g. Among the cultivars, ‘Pachamama’ showed the highest yield, with an average of 421.2 ± 52.99 g of dry flowers per plant, followed by ‘Mariquita’ with 212.98 ± 26.33 g, and ‘Conicet’ with 169.17 ± 16.88 g. Chromatographic analyses confirmed the distinct cannabinoid profiles of each cultivar, as expected (Table 4). Total cannabinoid yields (potency) in dried flowers range from 71 to 121 mg/g with ‘Conicet’ showing the lowest potency and ‘Mariquita’ the highest. Medicinal oils prepared from these cultivars achieved similar cannabinoid ratios and potencies to those observed in the flowers, with the highest concentrations in ‘Mariquita’ derived oils and the lowest in those from ‘Conicet’. Cannabinoid extraction efficiencies were between 7 and 14% (Table 4).

**Table 4:** Cannabinoid concentrations (THC: Δ⁹-tetrahydrocannabinol; CBD: cannabidiol) and yields in dried inflorescences and derived medicinal oils obtained in the present field experiment. Values correspond to a single composite sample per cultivar and a single analytical determination, prepared by pooling inflorescences from the main stem of ten plants grown under outdoor conditions

Cultivar	Dried flowers				Medicinal oils			
	**%THC** **(g/100 g)**	**%CBD** **(g/100 g)**	**Potency** **(mg/g)**	**Potency** **(%)**	**THC** **(mg/ml)**	**CBD** **(mg/ml)**	**Yield** **(mg/g flower)**	**Yield** **(%)**
*Conicet*	7.1	0	71.0	7.1	7.6	0	68.4	6.8
*Mariquita*	3.5	8.6	121.0	12.1	2.5	13.2	141.3	14.1
*Pachamama*	0.6	10.7	113.0	11.3	0.0	12.2	109.8	11.0

### Safety of flowers and medicinal oil

Neither total coliforms nor *E. coli* were detected in any of the dry flower or medicinal oil samples, with all values falling below the limit of quantification (Table 5).

**Table 5:** Heavy metal concentrations (As: arsenic; Cd: cadmium; Co: cobalt; Cr: chromium; Cu: copper; Ni: nickel; Pb: lead; Zn: zinc) and microbial counts (including total coliforms and *Escherichia coli*) in cannabis inflorescences and medicinal oil. Values are expressed as mean ± standard error (SE) (*n* = 3). Loq: limit of quantification (heavy metals: 0.20 mg/kg for solid samples and 0.002 mg/L for liquid samples; microorganisms: 1 MPN/g for inflorescences and 1 MPN/100 mL for oil). MPN: most probable number

**Cultivar**		**Total coliforms**	***E.coli***	**Heavy metal concentrations**																			
				**As**	**Cd**	**Co**			**Cr**			**Cu**			**Ni**			**Pb**			**Zn**		
*Conicet*	*Dry flowers*	*<Loq*	*<Loq*	*<Loq*	*<Loq*	0.42	±	0.03	0.76	±	0.1	16.7	±	0.43	0.99	±	0.05	0.68	±	0.13	54.94	±	3.69
	*Medicinal oil*	*<Loq*	*<Loq*	*<Loq*	*<Loq*	*<Loq*			*<Loq*			0.48	±	0.01	<Loq			<Loq			2.11	±	1.01
*Mariquita*	*Dry flowers*	*<Loq*	*<Loq*	*<Loq*	*<Loq*	0.34	±	0.08	0.66	±	0.11	15.71	±	2.02	1.18	±	0.25	0.89	±	0.14	55.19	±	8.85
	*Medicinal oil*	*<Loq*	*<Loq*	*<Loq*	*<Loq*	*<Loq*			*<Loq*			0.61	±	0.13	<Loq			<Loq			2.05	±	0.43
*Pachamama*	*Dry flowers*	*<Loq*	*<Loq*	*<Loq*	*<Loq*	*<Loq*			0.53	±	0.04	16.37	±	0.4	0.74	±	0.06	1.07	±	0.15	55.9	±	4.35
	*Medicinal oil*	*<Loq*	*<Loq*	*<Loq*	*<Loq*	*<Loq*			*<Loq*			0.55	±	0.08	<Loq			<Loq			1.25	±	0.61

Regarding the concentration of regulated heavy metals in dried flowers, arsenic (As) and cadmium (Cd) were below the limit of quantification in all three cultivars analyzed (< Loq), with values lower than the maximum limits established by the California Department of Cannabis Control. In contrast, lead (Pb) showed an average concentration of 0.88 mg/kg, with the highest values observed in ‘Pachamama’ and the lowest in ‘Conicet’, exceeding the maximum allowable limit established by California regulations (Table 5). With respect to the other elements analyzed that are not regulated under these standards, Cobalt (Co) was also below the limit of quantification in ‘Pachamama’ but was detectable in ‘Conicet’ and ‘Mariquita’, with concentrations of 0.42 and 0.34 mg/kg, respectively. Chromium (Cr) showed a mean concentration of 0.65 mg/kg across cultivars, with the highest values in ‘Conicet’ and the lowest in ‘Pachamama’. Zinc (Zn) and copper (Cu) presented the highest concentrations, with mean values across cultivars of 55.34 and 16.2 mg/kg, respectively. The highest Zn concentrations were recorded in ‘Pachamama’, while the highest Cu levels were found in ‘Conicet’. Nickel (Ni) showed a mean value of 0.97 mg/kg, with the highest concentration in ‘Mariquita’ and the lowest in ‘Pachamama’ (Table 5).

In medicinal oils, As, Cd, Co, Cr, Ni, and Pb concentrations were below the limit of quantification in all three cultivars (< Loq). Only Cu and Zn were detected, with mean concentrations across cultivars of 0.54 and 1.80 mg/L, respectively. The highest Cu concentration was found in the oil of ‘Mariquita’, while the oil from ‘Conicet’ showed the highest Zn levels (Table 5).

## Discussion

In this work, we analyzed the feasibility of medicinal cannabis cultivation under outdoor conditions using municipal reclaimed wastewater. A study conducted in Canada showed that outdoor cannabis cultivation can emit up to 50 times less carbon than indoor production, making it a highly desirable alternative from an environmental and economic perspective (Desaulniers Brousseau et al. 2024a). However, outdoor production is also associated with relevant limitations, including greater variability in chemical composition, reduced environmental control, and the seasonality of production, which may affect yield stability and cannabinoid profiles (Zandkarimi et al. 2023; Thawonkit et al. 2025). Additionally, outdoor grown cannabis has been found to contain fewer oxidized and degraded cannabinoids, as well as greater diversity and concentrations of terpenes compared to indoor-grown cannabis from the same genetics (Zandkarimi et al. 2023). Moreover, an analysis based on data from the Canadian industry revealed that the cost of producing 1 kg of cannabis in outdoors is six times lower than that of indoor production (Desaulniers Brousseau et al. 2024a). In this context, the possibility of outdoor cultivation using reclaimed water thus represents an opportunity for the development of the cannabis industry in arid regions, but only when appropriate agronomic management and quality control strategies are implemented. Cannabis is a species with a high capacity to accumulate heavy metals and other potentially toxic compounds (phytoaccumulation) (Zielonka et al. 2020; Golia et al. 2023), therefore, even when treated wastewater is not originated from industrial sources, it is essential to evaluate its chemical quality to ensure the safety of cannabis products produced with such irrigated water, especially when intended for human consumption. Due to the recent legalization of medicinal cannabis in Argentina, there is still no specific regulation establishing maximum concentrations of heavy metals or microorganisms in cannabis cultivation and its derivatives. In contrast, other countries in the region such as Chile, Uruguay, Colombia, and Ecuador apply safety standards and guidelines based on the European Pharmacopeia and the World Health Organization. In North America and Canada, specific legislation has been developed establishing concentration limits for heavy metals such as As, Pb, Cd, and Hg. The state of California, for instance, has one of the most stringent regulatory frameworks, with maximum limits in inhalable products set at 0.2 mg/kg for cadmium (Cd) and arsenic (As), and 0.5 mg/kg for lead (Pb). For edible cannabis products, the limits are 0.5 mg/kg for Cd and Pb, and 1.5 mg/kg for As (California, Department of Cannabis Control 2025).

### Soil and water physicochemical and microbiological properties

When soil contamination with heavy metals (including As, although it is technically a metalloid) occurs, whether due to agricultural practices or other human activities, these elements tend to accumulate in the upper soil layers, from where they can leach into groundwater or reach surface water, and can be absorbed by plants (Alloway 2013). In Argentina, soil quality guidelines are established by Regulatory Decree 831/93 of National Law 24,051 on hazardous waste, which defines threshold levels for different land uses (agricultural, residential, and industrial). Our results indicate that the soil used in this study is suitable for agricultural production, with initial concentrations of heavy metals between one and two orders of magnitude below the thresholds established for agricultural soils by current legislation. In our irrigation experiment, the continued use of treated wastewater (13,500 L applied over a 90-day crop cycle) did not significantly increase the concentration of metals in the soil. Final values at harvest were similar to the initial measurements. These results indicate that the contribution of heavy metals by reuse water to the soil is not significant, at least in the volumes of water used during a short crop cycle.

Heavy metals must be bioavailable for plants to be able to incorporate from the soil solution. The bioavailability of heavy metals depends on soil type (e.g., texture) and its physicochemical properties (Reichman 2002; Adamczyk-Szabela and Wolf 2022). Among these factors, soil pH plays a key role, as it affects the solubility and mobility of most metals. With exceptions (Mo, Se, and As), an increase in soil pH generally leads to a decrease in metal bioavailability (Reichman 2002; Silveira et al. 2003; Adamczyk-Szabela and Wolf 2022). In our study, soil pH values were not altered by the application of treated wastewater throughout the experiment. The soil maintained its slightly alkaline character, which is typical of soils in the semiarid Patagonian region (Rostagno et al. 1991; Bouza et al. 2007). Under these pH conditions, it is expected that most metals are present as precipitated forms, such as insoluble hydroxides, carbonates, or inorganic complexes, thus reducing their bioavailability (Alloway 2013; Xu et al. 2022). Electrical conductivity (EC) of the soil was also not significantly affected by the use of reclaimed water, remaining within the non-saline range throughout the crop cycle (Ismayilov et al. 2021).

The physicochemical characteristics of the treated wastewater used in this study are consistent with expected values for urban effluents subjected to secondary treatment processes (U.S. Environmental Protection Agency 2010). Mean pH values above 7, both in water and in soil, could potentially pose limitations to plant growth (Barrow and Hartemink 2023; Hartemink and Barrow 2023). However, no issues related to plant development or symptoms of nutrient deficiency were observed during the crop cycle. This may be explained by the fact that nutrient availability to plants results from a combination of pH effects on soil sorption processes and pH effects on plant uptake mechanisms (Barrow and Hartemink, 2023). The electrical conductivity (EC) of 1,583 μS/cm classifies the water as slightly saline (FAO 1992), which may impose some restrictions depending on the crop’s sensitivity. However, numerous cannabis varieties with salt tolerance have been reported (Huaran et al. 2019; Akram et al. 2021; Zhang et al. 2021).

Dissolved oxygen (DO) levels at the time of refill of the three irrigation tanks were relatively low, likely due to the high organic load of the effluent, as reflected in the elevated BOD₅ and COD values, as well as in the abundance of volatile suspended solids. In lagoon treatment systems, oxygen is supplied by the photosynthetic action of the microalgae growing there and, to a lesser extent, by wind action. In parallel, DO is used for respiration and the degradation of organic matter by microorganisms, corresponded in our study with the recording of a negative correlation between DO and COD concentrations; emphasizing that this has been magnified due to the storage of irrigation water in closed tanks without exposure to sunlight. In oversaturated systems, oxygen consumption may equal or exceed oxygen generation (Faleschini et al. 2012; Ragush et al. 2015).

Regarding nutrients, the water supplied nitrogen in the form of NH_4_^+^ (approximately 10 mg/L), as well as NO_3_^−^ and NO_2_^−^, and contributed with 13 mg/L of PO₄^3^⁻. Although nutrient concentrations in the soil were not directly measured, this decision was related to the nature of the experimental plot, which was established using locally sourced filling soil rather than a stabilized pedogenetic profile. Under these conditions, soil nutrient levels are highly variable and not necessarily representative of long-term site properties. Nevertheless, the cumulative nutrient input provided by irrigation water throughout the crop cycle was sufficient to support full plant development. In this context, reclaimed water functioned not only as an irrigation source but also as a relevant contributor of nutrients, aligning with the dual-use approach of these effluents in integrated water and nutrient management strategies (Pedrero et al. 2010).

The presence of microorganisms such as total coliforms or *E. coli* serves as an indicator of potential contamination (Chen et al. 2023). In the case of soil, although there are no regulations in Argentina establishing maximum microbial loads for agricultural soils, sampling at both the beginning and end of the experiment revealed only very low values of total coliforms, with no detection of *E. coli*, indicating the absence of fecal contamination throughout the crop cycle. Regarding the microbiological quality of the treated water, the results showed a sustained decrease in total coliforms and *E. coli* over the storage time in tanks or reservoirs. Similar reductions in fecal indicator bacteria during reclaimed water storage and the absence of *E. coli* in irrigated soils have been reported in studies on other crops, highlighting the effectiveness of storage and management practices in reducing microbiological risks associated with reclaimed water irrigation (Guo et al. 2017; Sunyer-Caldú et al. 2022).The rapid decrease registered may be associated with the high summer temperatures characteristic of the region during those months, which directly promote bacterial inactivation through thermal stress inside the tanks and indirectly, generating a rapid modification of the redox potential, going from widely positive values ​​at the beginning of filling to values ​​below −283 mV. (Nyieku et al. 2020; Wang et al. 2021). Correlations between total coliforms and *E. coli*, as well as environmental variables such as BOD₅, and total and volatile suspended solids suggest that the availability of organic matter could also be contributing to microbial decline in storage tanks (Kinnaman et al. 2012; Seo et al. 2019).

From a regulatory perspective, *E. coli* concentrations at tank refilling exceeded the WHO guideline value for the use of treated wastewater in crops with direct contact with irrigation water (≤ 1,000 MPN/100 mL; WHO 2006). However, after storage in the tanks, levels fell below this threshold. In the third refill event, where initial *E. coli* concentrations were particularly high, an additional sampling was conducted three days after refilling to better capture early dynamics. This sampling confirmed a marked reduction in *E. coli* concentrations over time. Notably, the rate of reduction observed after the first and second refill events was faster, with values decreasing more rapidly within the first 10 days of storage. It should be noted that, during the third refill event, *E. coli* concentrations were not assessed at day 10, as sampling efforts were focused on capturing early decay dynamics under high initial contamination conditions. While this approach provided valuable information on short-term microbial reduction, it limited direct comparability with the 10-day assessment performed in the first two events. Future studies should maintain consistent monitoring at this time point to confirm whether a 10-day storage period reliably ensures compliance with recommended microbiological thresholds under varying contamination scenarios. The values recorded after 10 days of storage following the first and second refill events, and after 30 days following the third refill event, were always below the threshold established for industrial crops (WHO 2006). This result highlights the importance of storage as a complementary treatment strategy to improve the microbiological quality of reclaimed water prior to its agricultural application (Jjemba et al. 2014; Sunyer-Caldú et al. 2022). Nevertheless, under the current operational conditions, the WHO guideline value was not met at the time of tank refilling in any of the evaluated events; therefore, compliance at the initiation of irrigation cannot be guaranteed. The variability observed between refill events indicates that bacterial contamination levels are not stable over time and reinforces the need for consistent microbiological monitoring. Additionally, as the present results correspond to a single cultivation cycle, further studies across multiple cycles are required to confirm the robustness of these findings across different operational conditions.

### Flower and cannabinoid yields

The three tested cultivars exhibited good development throughout the crop cycle, and flower yields were within the expected range reported in other outdoor production systems with similar production periods (90 days), (Desaulniers Brousseau et al. 2024a), although such comparisons should be interpreted cautiously due to differences in climatic conditions, soil characteristics, and plant material.

Treated wastewater was sufficient to supply nutrients during the entire growing period without the need for supplemental fertilization. This not only reduced the environmental footprint typically associated with outdoor cannabis production (Desaulniers Brousseau et al. 2024a, 2024b), but also resulted in savings on high-cost fertilizers. Although absolute cannabinoid concentrations obtained in this work were lower than the reference values reported for each cultivar during official registration, the characteristic chemotype of each cultivar was conserved under the experimental conditions. Differences between registered values and those observed in this study are likely related to contrasting cultivation conditions, particularly outdoor versus indoor environments, as well as the shorter flowering and production cycle (Janatová et al. 2018; Backer et al. 2019).

### Chemical and microbiological characteristics of inflorescences and cannabis oils

All heavy metals analyzed, such as As, Cd, Co, Cr, and Pb, were found at low levels (< 1 mg/kg) or non-detectable in flowers. The highest values were observed for Cu and Zn, which are essential nutrients in plant tissues and are commonly found at these concentrations (Lilay et al. 2024; Figas et al. 2025). Lead (Pb) concentrations in flowers, although low (0.8 ± 0.3 mg/kg) slightly exceeded the current regulatory limit established for inhaled flower products (0.5 mg/kg, California legislation). Given that Pb levels in soil remained constant throughout the cultivation cycle and were below the detection limit in the irrigation water, the accumulation of this metal in the plant material could be attributed to its presence in the soil and the previous described phytoaccumulation capacity of cannabis for heavy metals (Golia et al. 2023). Therefore, if the intended use of the product is for inhalation, special attention should be given to substrate quality and heavy metal concentrations, particularly Pb, in order to avoid contamination and ensure the safety and compliance of the final product. It should also be considered that the bioaccumulation capacity of cannabis may vary among cultivars (Milan et al. 2024), thus, additional testing is recommended when varieties different from those evaluated in this study are used.

As for the medicinal oils, the concentrations of all toxic elements were below the regulatory thresholds and, in all cases, below the method’s quantification limit (0.020 mg/kg), indicating that the use of flowers irrigated with treated municipal wastewater is suitable for the production of medicinal cannabis oil.

It should be noted that, while this study focused on heavy metals and selected microbiological indicators as key safety parameters, other potential contaminants associated with reclaimed water reuse, such as contaminants of emerging concern (CECs), including pharmaceuticals, personal care products, and endocrine-disrupting compounds were not assessed. Previous studies in other crops irrigated with reclaimed water have shown variable uptake of these compounds depending on water quality, soil properties, and crop species (Sunyer-Caldú et al. 2022; García-Valverde et al. 2024). Although data for *Cannabis sativa* L. remain scarce, these findings highlight the importance of considering a broader range of potential contaminants in future studies, particularly for medicinal applications.

### Conclusions

In this work, we assessed the feasibility and safety of cultivating outdoor *Cannabis sativa* L. using reclaimed municipal wastewater with physico-chemical and microbiological characteristics comparable to those evaluated in the present study. Our results support the viability of the system, with favorable growth conditions and promising yields, although careful monitoring of microbiological and heavy metal parameters remains essential to ensure regulatory compliance under operational conditions. Maintaining microbiological concentrations within established thresholds is critical to protect environmental and public health. Enhanced monitoring strategies and/or extended retention times prior to irrigation may be necessary to align with international standards for unrestricted agricultural reuse. On-farm storage before irrigation could also provide an additional safety buffer under high contamination scenarios. These findings indicate that, under appropriate agronomic management, including controlled irrigation practices, adequate storage of reclaimed water to improve microbiological quality, and consideration of local soil and environmental conditions, reclaimed wastewater can represent a viable resource for medicinal cannabis cultivation in arid and semi-arid regions. Future studies should incorporate a broader assessment of potential contaminants to further consolidate the safe use of reclaimed water in medicinal cannabis production systems.

## Data Availability

The datasets generated and/or analyzed during the current study are available from the corresponding author on reasonable request.
